# Three-Way k-Means Model: Dynamic Optimal Sensor Placement for Efficient Environment Monitoring in Pig House

**DOI:** 10.3390/ani14030485

**Published:** 2024-02-01

**Authors:** Haopu Li, Bugao Li, Haoming Li, Yanbo Song, Zhenyu Liu

**Affiliations:** 1 College of Agricultural Engineering, Shanxi Agriculture University, Jinzhong 030801, China; lihp0307@163.com; 2 College of Animal Science, Shanxi Agriculture University, Jinzhong 030801, China; jinrenn@163.com; 3 College of Information Science and Engineering, Shanxi Agriculture University, Jinzhong 030801, China; lihaoming@163.com; 4 College of Life Sciences, Shanxi Agriculture University, Jinzhong 030801, China; syblzy@126.com

**Keywords:** dynamic sensor placement, pig house, three-way k-means, particle swarm optimization, genetic algorithm

## Abstract

**Simple Summary:**

The focus of pigsty environmental monitoring is to deploy an appropriate number of sensors in optimal locations. Therefore, researching the optimal placement of sensors inside pigsties is of great significance. This study, through a clustering analysis of environmental data collected by sensors in pigsties, has devised a seasonal dynamic sensor layout, providing a feasible solution for optimizing the internal environmental monitoring of pigsties. This will offer more rational and reliable environmental monitoring data for the control system within pigsties, reducing the number of sensors and minimizing data redundancy. The results indicate that placing a smaller number of sensors at key locations inside pigsties can effectively monitor the internal environment.

**Abstract:**

Sensors were of paramount importance in the context of poultry and livestock farming, serving as essential tools for monitoring a variety of production management parameters. The effective surveillance and optimal control of the swine facility environment critically depend on the implementation of a robust strategy for situating the optimal number of sensors in precisely the right locations. This study presents a dynamic sensor placement approach for pigsties using the three-way k-means algorithm. The method involves determining candidate sensor combinations through the application of the k-means algorithm and a re-clustering strategy. The optimal sensor locations were then identified using the Joint Entropy-Based Method (JEBM). This approach adjusts sensor positions based on different seasons (summer and winter) to effectively monitor the overall environment of the pigsty. We employ two clustering models, one based on particle swarm optimization and the other on genetic algorithms, along with a re-clustering strategy to identify candidate sensor combinations. The joint entropy-based method (JEBM) helps select the optimal sensor placement. Fused data from the optimal sensor layout undergo a fuzzy fusion process, reducing errors compared to direct averaging. The results show varying sensor needs across seasons, and dynamic placement enhances pigsty environment monitoring. Our approach reduced the number of sensors from 30 to 5 (in summer) and 6 (in winter). The optimal sensor positions for both seasons were integrated. Comparing the selected sensor layout to the average of all sensor readings representing the overall pigsty environment, the RMSE were 0.227–0.294 and the MAPE were 0.172–0.228, respectively, demonstrating the effectiveness of the sensor layout.

## 1. Introduction

Pig houses and other livestock structures were designed to create optimal growth conditions for the animals they house. Pigs, being homeothermic creatures, were highly sensitive to variations in the temperature and humidity in their surroundings. An abnormal environment with temperature and humidity levels may trigger heat stress in pigs, potentially resulting in fatalities, particularly in severe cases [1,2]. Traditionally, the internal environment of livestock structures has been monitored through one or more sensors. However, pinpointing the most suitable sensor positions to holistically represent the internal environment remains a challenge [3,4]. Furthermore, the heat generated by the animals themselves, coupled with variations in ventilation locations and the insulation efficacy within livestock structures, introduces considerable disparities in the internal environment. Hence, to facilitate the rational and efficient monitoring of the pig house’s internal conditions, the selection of sensor positions that accurately reflect the overall environment within the structure is imperative.

In pig houses, the conventional approach, informed by empirical wisdom, is to situate sensors at the central location of the facility with the assumption that it adequately represents the overall indoor environment. However, in practicality, relying solely on the central position as a proxy for the entire indoor environment proves insufficient. Yeo et al. [5] substantiated, through modeling and on-site experiments, that variations exist within different zones of the pig house environment, contingent upon the operational output of the Heating, Ventilation, and Air Conditioning (HVAC) system. The misplacement of sensors at inappropriate locations can lead to erroneous data collection, consequently resulting in the mismanagement of the control system and the compromise of the conducive rearing environment within the pig house. Furthermore, the placement of sensors at strategic locations holds pivotal importance for the twin goals of energy conservation and emissions reduction, as it helps avert unnecessary energy consumption [6].

In tackling the challenge of the optimal sensor placement, researchers have harnessed a diverse array of algorithms and techniques for sensor configuration, encompassing genetic algorithms [7,8], heuristic algorithms [9], optimization algorithms [10], information theory [11,12], and machine learning [13]. This study is motivated by the objective of exploring the optimal positioning of temperature and humidity sensors within pig houses, with the overarching aim of orchestrating their layout in a manner that is both effective and cost-efficient for comprehensive indoor environment monitoring.

In the context of genetic algorithm-based sensor placement, previous research has explored various strategies. For example, Hu et al. [14] used a dual-structure coding genetic algorithm to optimize sensor combinations in chicken houses, using a grey correlation degree-based objective function. Ajani et al. [15] introduced a genetic programming-based method for optimal sensor placement in greenhouse environmental monitoring and control. Furthermore, Zhang et al. [16] introduced a novel genetic algorithm called Meta-GA, which incorporates a competition group mechanism and gene pool. They used Meta-GA with experimental data to construct a finite element model that considers the parameter randomness and initial damage for optimal sensor placement.

In the realm of the optimal sensor placement driven by optimization algorithms, Matsuda et al. [17] introduced an innovative approach for addressing the optimal sensor placement problem in high-dimensional systems, leveraging the simulated annealing mechanism. Meanwhile, Zhang et al. [18] developed a Bayesian probabilistic virtual sensor model that accounts for model uncertainty and measurement errors. They utilized the K-L divergence to derive an unbiased estimate of modal parameters and introduced a novel variance determination technique using the regularization mechanism inherent in Bayesian methods. Their approach incorporated the NSGA-II multi-objective optimization algorithm to derive the ultimate optimal sensor placement solution.

In the domain of optimal sensor placement relying on clustering algorithms, Wang et al. [19] harnessed the k-means algorithm to ascertain both the quantity and positions of sensors. Their innovative contribution was the introduction of a re-clustering strategy, which served to enhance the efficacy of k-means clustering results and elevate the overall monitoring efficiency of sensor deployment. Meanwhile, Uyeh et al. [20] applied the k-means++ algorithm to facilitate optimal sensor placement within a greenhouse setting. Their approach entailed optimizing the initial 56 monitoring locations by utilizing the average silhouette method for the selection of the optimal number of sensors. Furthermore, in addressing optimal sensor placement challenges, Lee et al. [21] integrated error-based and entropy-based methods for greenhouse sensor placement. This approach identifies optimal sensor locations to effectively capture the entire facility’s environmental conditions and detect temperature variations. In a different approach, Guo et al. [22] introduced a sensor placement technique using reinforcement learning. They built a pre-learning library with Proper Orthogonal Decomposition (POD) and employed Q-learning to select the best sensor configuration for quality data reconstruction.

However, in the existing sensor layout schemes proposed, although various algorithms have been employed to optimize sensor placement, identifying critical positions and optimal quantities within livestock and poultry facilities, most of them overlook the influence of seasonal variations. Additionally, a majority of studies only utilize a single algorithm for layout optimization without setting up multiple models for comparison in experimental facilities. As a result, there were still some shortcomings and areas for improvement.

In this study, we conducted a layout analysis of internal sensors in pigsties, utilizing the three-way k-means algorithm, optimization algorithms, and the joint entropy-based method to analyze monitoring data within pigsties. This research aims to determine the optimal number and positions of sensors. The study provides technical support for the intelligent monitoring of livestock and poultry housing. The rest of this paper is organized as follows. In Section 2, we introduce the overall research process and the experimental pig houses. Section 3 describes the experimental comparison and analysis. Section 4 describes the main contributions of the study as well as its shortcomings. Finally, Section 5 concludes this work.

## 2. Materials and Methods

### 2.1. Description of the Experimental Pig House

**Figure 1:**
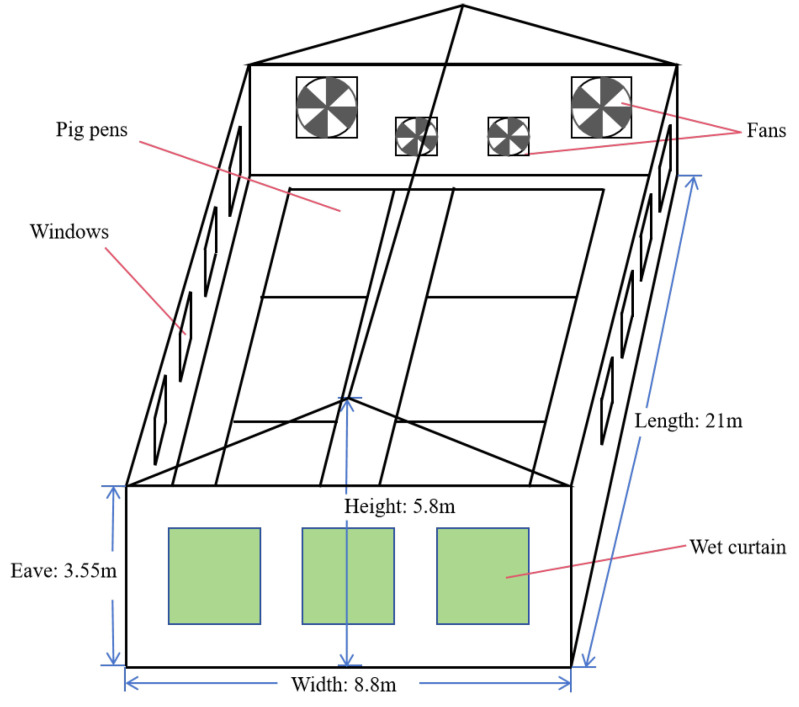
Schematic diagram of experimental pig house.

The experimental pig house was situated in Fenyang City, Shanxi Province, China, within the geographic coordinates of E111°26′–E112°00′, N37°08′–N37°29′. The dimensions of the experimental pig house were as follows: it measured 21 m in length, 8.8 m in width, and stood at a height of 3.55 m. The walls of the pig house were constructed using 240 mm thick brick walls, plastered both internally and externally, as depicted in Figure 1. All sides of the pig house exhibited uniform wall materials and thickness. The windows were composed of a single-layer glass, plastic, and steel frame. Each of the south and north longitudinal walls featured a total of seven windows, with each window measuring 1.5 m in both length and width. The lower edge of these windows was situated 0.7 m above the ground inside the house.

Internally, the layout of the pig house comprised double-row pig pens and a single-row aisle, with the aisle measuring 1.37 m in width. Within the pig house, there were a total of 14 pig pens, each measuring 2.86 m × 3.71 m with a height of 1 m. Beneath each slatted floor, there were four manure pits with a depth of 0.7 m, which were cleared out using a plug-type flushing method. The pig house was equipped with one door, measuring 2.5 m in height and 1.23 m in width. The design of the experimental pig house catered to the accommodation of 158 finishing pigs, with the total floor area of the pig pens spanning 148.55 m². Each finishing pig occupies a floor area ranging from 0.8 m² to 1.2 m². The pig breeds included Large White and Yorkshire, and they were approximately 7 months old, with an average body weight of 70–80 kg per pig.

The roof of the pig house was constructed using double-sloped color steel sandwich panels with a thickness of 100 mm, and the pig house featured a 2.25 m high single-layer color steel ceiling. Various ventilation and cooling equipment were installed within the pig house, including fans, wet curtains, a lighting system, and an automatic control system. The pig house was oriented in a north-to-south direction, with the wet curtains positioned on the east wall and the fans on the west wall. The wet curtains had dimensions of 1.83 m in width, 1.9 m in height, and a thickness of 0.15 m. The bottom of the wet curtains was positioned at a height of 0.68 m above the ground inside the house. A total of four fans were installed, comprising two different models. The diameter of the large fan was 1.08 m, while the diameter of the small fan was 0.76 m; both fans belonged to the axial fan type. The large fan, under operational conditions, had a pressure difference of 45 Pa and a mass flow of air of 3500 m^3^ per hour. It was positioned at heights of 0.55 m and 0.63 m above the indoor floor. The small fan, under operational conditions, exhibited a pressure difference of 20 Pa and a mass flow of air of 1500 cubic meters per hour. It was situated at heights of 0.85 m and 0.93 m above the indoor floor.

Regarding the operation of the ventilation and cooling system, the airflow rate was regulated according to the deviation in the air temperature concerning the specified target air temperature. If the air temperature surpasses the designated threshold, the ventilation fans operate at their maximum performance (maximum ventilation). Conversely, when the temperature falls below the target air temperature, the ventilation fans function in a low-performance setting (minimum ventilation).

### 2.2. Description of the Environmental Monitoring

**Figure 2:**
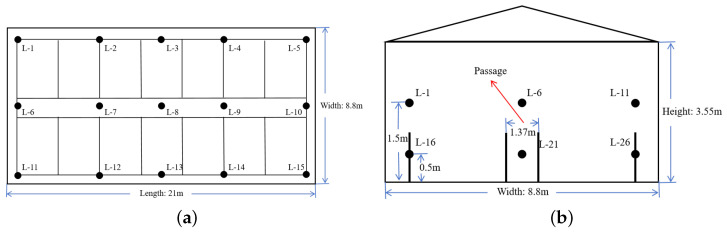
Initial locations of measurement sensor: (**a**) Top view; (**b**) Left view.

The control of the environmental conditioning unit within the experimental pig house primarily relies on temperature and humidity data, which were monitored by the HC2S3 sensor. (Campbell Scientific Inc., Logan, UT, USA) These data inform the operation of essential equipment within the pig house, including fans, wet curtains, and heaters. The HC2S3 sensor was powered by a direct current (DC) supply of 9–18 V. It had a temperature measurement range of −40 to 80 °C with an accuracy of ±0.1 °C. The humidity measurement range was from 0 to 100%RH with an accuracy of ±0.8%RH. In the experimental pigsty, the selection of sensors was based on a comprehensive consideration of accuracy, durability, communication capability, and cost. The current sensors used an exhibited accuracy that meets the requirements for the daily management in the pigsty. They were constructed with waterproof, dustproof, and shock-resistant materials, ensuring reliable durability. Additionally, these sensors featured Wi-Fi wireless communication functionality, and their cost was acceptable. To facilitate the real-time monitoring of environmental fluctuations, primarily concerning temperature and humidity, a total of 30 temperature and humidity sensors were strategically positioned within the experimental pig house. These sensors were organized into two layers: the upper layer was situated 1.5 m above the ground, while the lower layer was positioned 0.5 m above the ground. Each layer comprised 15 sensors, evenly distributed with 5 measuring points along the horizontal axis and 3 along the vertical axis (refer to Figure 2a,b for a visual representation). Importantly, these sensors had no adverse impact on the regular activities of the pigs and were well-suited for installation within the animal living area of livestock buildings. The collected air temperature and relative humidity data were recorded at 10 min intervals and stored using a multi-channel data collection device (BN-BR001BYBF, Bourne Inc., Beijing, China).

### 2.3. Optimal Sensor Placement

**Figure 3:**
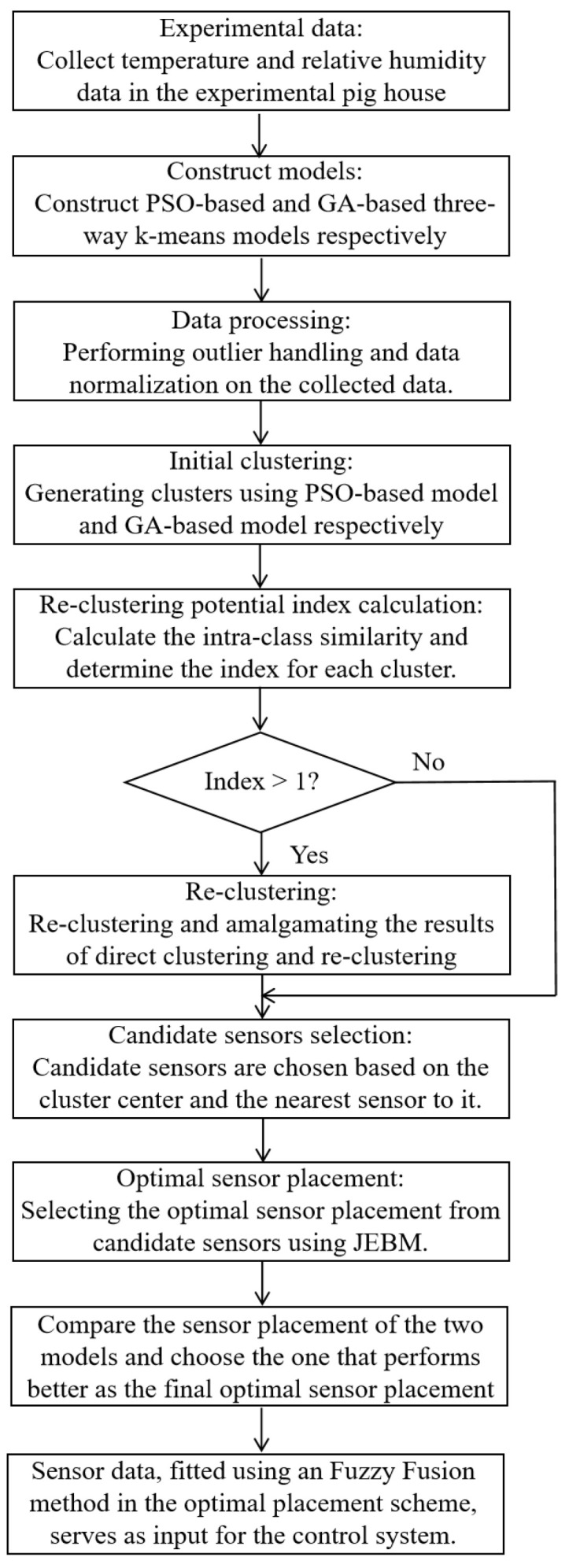
Research flow to determine optimal sensor placement.

Sensor placement within the pigsty involves a delicate balance between monitoring efficiency and economic considerations. The methodology employed in this study had been outlined in Figure 3). The experimental data were collected over the course of a year, from September 2021 to September 2022, with readings taken every 30 min, resulting in a total of 17,520 data points (17,520 = 365 × 24 × 2). These measurements include air temperature and relative humidity from 30 distinct locations within the pigsty. Based on the data collected, sensor installation locations were determined according to specified quantities, and a fuzzy fusion method was applied to combine and process the data acquired by the sensors. This allowed for environmental control within the pigsty during all seasons of the experimental stage, including spring, summer, autumn, and winter.

The focus of this study was to reduce redundant sensors, thereby lowering computational costs and improving efficiency. Therefore, we opted for the simple and efficient three-way k-means algorithm. Considering the significant impact of initial cluster centers on the clustering results in the k-means algorithm, we improved it using particle swarm optimization and genetic algorithms, both possessing strong global search capabilities. This enhancement aimed to better identify suitable cluster centers and enhance the overall clustering performance. The workflow of this study was summarized as follows:

Data Preprocessing. Initially, the data undergo comprehensive preprocessing, encompassing procedures such as outlier management and data normalization.

PSO-based three-way k-means model: We construct a three-way k-means model based on the PSO algorithm. The data were initially subjected to clustering using this model, resulting in the division of sensors into multiple clusters. The process commences with two clusters, and the maximum number of clusters was tailored to the specific conditions of the pigsty. In this particular study, we explore clustering numbers ranging from 2 to 8.

GA-based three-way k-means model: An additional three-way k-means model was established based on the GA. This model utilizes a genetic algorithm to ascertain the optimal number of clusters. Furthermore, the intra-class similarity was computed for each cluster, yielding a re-clustering potential index. Clusters exhibiting an index exceeding 1 were subject to further re-clustering.

Combining clustering results: The ultimate clustering outcome was achieved by integrating the results derived from the two distinct clustering models.

Selection of candidate sensors. Within each cluster, we choose both the cluster center and the sensor in closest proximity to the cluster center as candidate sensors.

Sensor combination. Candidate sensors were selected within each cluster for potential sensor combination. This approach markedly reduces the computational complexity and enhances the operational efficiency in comparison to employing a brute-force approach.

This comprehensive approach not only optimizes the placement of sensors but also considerably enhances the computational efficiency. It ultimately leads to the selection of an optimal sensor configuration, promoting efficient and reliable data collection for precise monitoring and control of the pigsty environment.

The selection of an optimal sensor placement was accomplished through the application of the joint entropy-based method (JEBM). Post clustering the sensors using specialized algorithms, a set of candidate sensors was derived by selecting both the cluster center and the sensor closest to the cluster center from each cluster. This method mitigated the issues associated with low efficiency and high computational costs that arose when using a brute force combination approach. For instance, in scenarios where the ideal number of sensors was 5, the brute force method resulted in a total of 142,506 potential sensor combinations, while the clustering process using the three-way k-means model narrowed it down to a more manageable 32 combinations.

To further refine the data quality and precision, the study employed the Fuzzy Fusion Method. This method facilitates the fusion of data collected by sensors in the optimal sensor layout scheme. It effectively mitigates the risk of significant errors caused by the direct averaging of sensor data. The fused data was subsequently integrated into the control system, serving as the input for the optimized control of the pigsty environment. This comprehensive approach not only refines the sensor selection process but also enhances the overall effectiveness and reliability of the control system within the pigsty.

### 2.4. Dividing the Data Set Based on Sample Stability

Li et al. [23] incorporated sample stability into the clustering ensemble and introduced a clustering ensemble grounded in sample stability. This section offers an in-depth examination of sample stability.

Suppose $X=\{x_{1},x_{2},...,x_{n}\}$ was a data set with samples, and $Q=\{q_{1},q_{2},...,q_{L}\}$ represents the set of clustering results obtained through *L* iterations of clustering using the same clustering algorithm with varying parameters or different clustering algorithms. We define $P_{ij}$ to represent the frequency with which samples $x_{i}$ and $x_{j}$ were assigned to the same cluster:

(1) $$P_{ij}=\frac{1}{L}\sum_{i=1}^{L}H\left(q_{l}\left(x_{i}\right),q_{l}\left(x_{j}\right)\right)$$

where,

(2) $$H\left(q_{l}\left(x_{i}\right),q_{l}\left(x_{j}\right)\right)=\left\{\begin{array}{cc} 1 & q_{l}\left(x_{i}\right)=q_{l}\left(x_{j}\right)\\ 0 & q_{l}\left(x_{i}\right)\neq q_{l}\left(x_{j}\right) \end{array}\right..$$

$q_{l}\left(x_{i}\right)$ represents the cluster to which the sample $x_{i}$ belongs in the *l*-th clustering result. Similarly, $q_{l}\left(x_{j}\right)$ represents the cluster to which the sample $x_{j}$ belongs in the *l*-th clustering result.

If the sample was assigned to the same cluster in the *l*-th cluster, then the value of $H(q_{l}\left(x_{i}\right),q_{l}\left(x_{j}\right))$ was 1, otherwise the value was 0.

After calculating the co-occurrence frequency between all samples, a relationship matrix *P* will be generated, which was used to represent the relationships between samples.

- When $P_{ij}$ was equal to 1, it means that samples $x_{i}$ and $x_{j}$ were assigned to the same cluster in multiple clusters, and the relationships between the two samples were stable.
- When $P_{ij}$ was equal to 0, it means that samples $x_{i}$ and $x_{j}$ were assigned to the different cluster in multiple clusters, and the relationships between the two samples were also stable.
- When $P_{ij}$ was greater than 0 but less than 1, it means that samples $x_{i}$ and $x_{j}$ were sometimes assigned to the same cluster and sometimes assigned to a different cluster in multiple clusters, thus the relationship between the two samples was unstable.

Expanding on the preceding analysis, stable relationships between two samples could be characterized by the following criteria: (1) a majority of clustering outcomes assign them to the same cluster, or (2) a majority of clustering outcomes assign them to different clusters. Consequently, depending solely on co-occurrence frequency proved inadequate for discerning sample relationships. In a prior investigation [24], a stability function was introduced as an assessment metric for discriminating among samples. This function employs the average confidence of the frequency between one sample and another as the stability measure. The stability calculation formula for a sample was as follows:

(3) $$s\left(x_{i}\right)=\frac{1}{n}\sum_{j=1}^{n}f\left(P_{ij}\right)$$

where $x_{i}$ denotes the *i*-th sample of dataset, *n* denotes the number of datasets, $P_{ij}$ represents the frequency with which samples $x_{i}$ and $x_{j}$ were assigned to the same cluster, $f(P_{ij})$ denotes the mapping function, and the calculation function was as follows:

(4) $$f\left(P_{ij}\right)=\left\{\begin{array}{cc} |\frac{P_{ij}-t_{0}}{t_{0}}| & P_{ij}<t_{0}\\ |\frac{P_{ij}-t_{0}}{1-t_{0}}| & P_{ij}\geq t_{0} \end{array}\right..$$

where $t_{0}$ was the threshold calculated using the maximum variance threshold method [25], and the calculation process was as follows:

Assuming the relationship matrix composed of the co-occurrence frequency $P_{ij}$ was $P=p_{1},p_{2},...,p_{w}$. We divide *P* into two parts $P_{0}$ and $P_{1}$, using a threshold $t_{0}$∈ (0,1).

(5) $$P_{0}=\left\{p_{i},p_{i}<t_{0},1\leq i\leq w\right\}$$

(6) $$P_{1}=\left\{p_{i},p_{i}\geq t_{0},1\leq i\leq w\right\}$$

Evaluate the performance of each threshold by calculating the inter-class variance, and select the most suitable threshold. The formula for calculating the inner-class variance $\sigma_{t}$ was as follows:

(7) $$\sigma_{t}=\omega_{0}{\left(\mu_{0}-\mu \right)}^{2}+\omega_{1}{\left(\mu_{1}-\mu \right)}^{2}$$

(8) $$\omega_{0}=\frac{|P_{0}|}{\left|P\right|},\omega_{1}=\frac{|P_{1}|}{\left|P\right|}$$

(9) $$\mu =\frac{\sum_{p_{i}\in P}p_{i}}{\left|P\right|},\mu_{0}=\frac{\sum_{p_{i}\in P_{0}}p_{i}}{|P_{0}|},\mu_{1}=\frac{\sum_{p_{i}\in P_{1}}p_{i}}{|P_{1}|}$$

By maximizing the inter class variance $\sigma_{t}$, the optimal threshold $t_{0}$ was obtained.

The procedure for splitting the data set into the kernel data set and the outer data set based on the sample stability was outlined as follows.

(1) Calculate the co-occurrence frequency by Formula (1), and then construct a relationship matrix *P*;
(2) Calculate the threshold $t_{0}$ of the relationship matrix using Formula (7) with *P* as input;
(3) Calculate the stability $s(x_{i})$ of each sample in the dataset using Formula (3) to obtain the stability set *S*;
(4) Calculate the stability threshold $s_{0}$ using Formula (7) with *S* as input;
(5) Kernel dataset $K\left(X\right)=\{x_{i}\in X|s\left(x_{i}\right)>s_{0}\}$; Outer dataset $R\left(X\right)=\{x_{i}\in X|s\left(x_{i}\right)\leq s_{0}\}$.

### 2.5. Three-Way k-Means Algorithm Based on PSO

#### 2.5.1. Particle Swarm Optimization

Particle Swarm Optimization (PSO) was an intelligent optimization algorithm inspired by avian flight and foraging behaviors. Each particle within the algorithm’s search space represents a potential solution to an optimization problem. The fitness of particles was determined by the optimization problem or objective function, assessing the individual particle quality through fitness comparisons. The algorithm was relatively straightforward, easy to comprehend and implement. It exhibits excellent global search capabilities, particularly in addressing problems within high-dimensional and complex spaces, garnering attention from scholars in the field [24].

The steps of PSO was as follows.

(1) Particle swarm initialization: Randomly generate *n* particles as the initial position of the particle swarm;
(2) Compute the fitness value for each particle using the fitness function. The fitness function employed in this study was based on the sum of squared errors, with the calculation formula outlined as follows:
  (10) $$f\left(x\right)=\sum_{i=1}^{k}\sum_{x_{j}\in C_{i}}d{\left(x_{j},C_{i}\right)}^{2}$$
  Among them, the criterion function f(x) was the total distance from the sample to the corresponding cluster center, which needs to be minimized. $d(x_{j},C_{i})$ was the distance from sample $x_{j}$ to the cluster center $C_{i}$, calculated as follows.
  (11) $$d\left(x_{j},C_{i}\right)=\sqrt{\sum_{t=1}^{k}{\left(x_{jt}-C_{it}\right)}^{2}}$$
  Among them, $x_{j}$ represents the *j*-th object, $C_{i}$ represents the *i*-th cluster center, $x_{jt}$ represents the *t*-th attribute of the *j*-th object, and $C_{it}$ represents the *t*-th attribute of the *i*-th cluster center.
(3) Find and update the individual extremum $pBest_{i}$ and global optimal value gBest for each particle, as well as their corresponding particle positions $P_{id}$ and $P_{gd}$;
(4) Update the present velocity and position of each particle by utilizing the following formula, which relies on its individual extreme value position and global optimal value position;
  (12) $$v_{i}\left(t\right)=\omega \times v_{i}\left(t-1\right)+\gamma_{1}\left(P_{id}-x_{i}\left(t\right)\right)+c_{2}\times \gamma_{2}\left(P_{gd}-x_{i}\left(t\right)\right)$$
  Among them, ω was an inertia weight, usually taken between [0.1, 0.9]. The larger the value, the stronger the global search ability and the weaker the local search ability. The smaller the value, the stronger the local search ability and the weaker the global search ability. $c_{1}$ and $c_{2}$ was the learning factor, also known as the acceleration constant, which was a parameter used to adjust the relative importance of $p_{id}$ and $P_{gd}$, usually $c_{1}$ = $c_{2}$ = 2. $\gamma_{1}$ and $\gamma_{2}$ represents a random number of (0, 1). $p_{id}$ represents the *d*-th dimension of the individual extreme value of the *i*-th variable. $P_{gd}$ represents the *d*-th dimension of the global extreme value of the *i*-th variable. $x_{i}\left(t\right)$ represents the position of the *i*-th particle in the *d*-th dimensional space, and the calculation formula was as follows:
  (13) $$x_{i}\left(t\right)=x_{i}\left(t-1\right)+v_{i}\left(t\right)\times T$$
  where *T* was usually taken as 1
(5) If the termination condition was met, the algorithm ends; otherwise, turn to step (2).

#### 2.5.2. PSO-Based Three-Way k-Means Algorithm

The concept behind the three-way k-means algorithm, which incorporates particle swarm optimization, entails an initial dataset division into two components: the high stability kernel data set K(X) and the low stability outer data set R(X) through a sample stability analysis. When initializing a particle swarm, *k* data points from the kernel data set K(X) were randomly selected as initial clustering centers. These centers were then assigned values to create the initial particles, thereby forming a particle swarm through multiple iterations. Utilizing the enhanced particle swarm optimization algorithm, the system iteratively seeks the optimal solution for the data set, which corresponds to the solution of the cluster center. Once the cluster center was determined, data points were assigned to the nearest cluster based on the nearest neighbor distance, calculated as shown in Formula (11). The resulting output was the data set’s clustering set.

To implement the algorithms in this study, Python 3.8 was selected as the programming language. We utilized PyCharm 2023.3.2 Community as the coding software and Origin 2021 for generating plots of the experimental data. The operating system was Windows 11, powered by a 12th Gen Intel(R) Core(TM) i5-12400F processor (Intel Corporation, Santa Clara, CA, USA) running at 2.50 GHz, with 16GB of RAM memory, and equipped with an Nvidia GeForce RTX 2070 SUPER graphics card (Colorful Group, Shenzhen, China). The steps of the PSO-based three-way k-means algorithm were shown in Algorithm 1. In the follow section, we will explain the steps of the algorithm in detail:
**Algorithm 1** PSO-based three-way k-means.**Input:** dataset *X*, cluster number *k*, inertial factor ω, learning factors $c_{1}$, $c_{2}$ **Output:** three-way k-means result $C=\{\left(C_{o}\left(C_{1}\right),F_{r}\left(C_{1}\right)\right),...,\left(C_{o}\left(C_{k}\right),F_{r}\left(C_{k}\right)\right)\}$
1:Divide the dataset *X* into kernel dataset K(X) and outer dataset R(X) by sample stability;2:Randomly select *k* samples as initial particles;3:Repeat the process *m* times to generate *m* particles;4:Initialize the particles’ velocity and position;5:Calculate the distances between all samples within each particle to the cluster centers using Formula (11), and partition the samples based on the nearest neighbor principle;6:Compute the fitness function value for each particle using Formula (10);7:Compare the fitness function value of each particle with the best fitness value it has encountered. If it is better than its current position, update the particle’s best position $P_{id}$;8:Compare the fitness values of all particles. If the best individual’s local best $P_{id}$ was better than the global best $P_{gd}$, update the global best to be $P_{id}$;9:Update the particle’s velocity and position using Formulas (12) and (13), respectively;10:Repeat steps 4 through 9 until a satisfactory position was achieved (or until reaching the maximum number of iterations);11:Calculate the Euclidean distance between samples ∈K(X) and cluster centers;12:Assign the samples to the core region of the cluster that was closest to their respective cluster centers;13:Calculate the Euclidean distance between samples ∈R(X) and cluster centers;14:Assign the samples to the boundary region of the cluster that was closest to their respective cluster centers;15:Output the three-way k-means result.


In step 1, employ the sample stability to partition the dataset into the kernel data set and the outer data set.

In steps 2–4, initialize the particle swarm. Randomly select k data objects from the kernel data set as the initial cluster centers, treating them as the initial particles. This process was iterated n times, generating n particles, each with initialized velocity and position.

In step 5, calculate the distances from all samples within each particle to the cluster centers using Formula (11). Following the principle of proximity, assign each sample to the cluster whose center was closest, thereby effectively partitioning them into their respective clusters.

In steps 6–8, Calculate the fitness function value for each particle using the Formula (10). Compare the fitness function value of each particle with the fitness function value of the best position it has encountered so far. If it is better than the current position, update the particle’s best position $P_{id}$. Compare the fitness values of all particles, and if the best individual value $P_{id}$ was superior to the global best value $P_{gd}$, update the global best value to $P_{id}$.

In step 9, Update the particle’s velocity and position by applying Equation (12) and Equation (13), respectively.

In step 10, iterate through the aforementioned steps from 4 to 9 until the particles attain an adequately optimal position or the algorithm reaches its maximum iteration count, upon which it terminates.

In steps 11–14, following the acquisition of k initial cluster centers, compute the Euclidean distance between the samples in the kernel data set and the initial cluster centers. Then, assign these samples to the core domains of the cluster that exhibits the closest proximity in terms of distance. Similarly, calculate the Euclidean distance between the samples in the outer data set and the initial cluster centers, subsequently assigning these samples to the boundary domains of the cluster that was nearest to them in terms of distance.

In step 15, output the cluster result.

### 2.6. Three-Way k-Means Algorithm Based on Genetic Algorithm

#### 2.6.1. Genetic Algorithm

To mimic the genetic processes observed in nature, an adaptive and globally oriented optimization search algorithm, known as the Genetic Algorithm, has been introduced [26]. This algorithm possessed robust global search capabilities, coupled with the ability to conduct localized searches. It was applicable to a diverse range of problems, facilitating parallel searches among multiple individuals to enhance efficiency. This algorithm began by encoding each potential solution as a chromosome. Randomly selecting a subset of chromosomes formed the initial population. Next, using a predefined fitness function, the fitness values of all chromosomes were computed. Subsequently, chromosomes with higher fitness values were selected as the new parent chromosomes. Through operations like crossover and mutation, offspring chromosomes with even higher fitness values were generated. After several generations of reproduction, the chromosome with the highest fitness value [27], representing the optimal solution to the problem, was obtained.

The steps of GA algorithm was as follows.

(1) Configure parameters including the number of individuals, crossover probability, mutation probability and termination conditions;
(2) Randomness initializes the code string in the parent group with an initial value;
(3) Set the evolution algebra to 0;
(4) Calculate the fitness values of each gene string;
(5) Conduct genetic operations to generate offspring based on specified probability parameters;
(6) Update the parent population with the offspring to produce the next generation of individuals;
(7) If the termination conditions have not been met, proceed to step (4). If they were satisfied, the optimal solution was achieved.

#### 2.6.2. GA-Based Three-Way k-Means Algorithm

This study incorporated a genetic algorithm with a three-way k-means algorithm to enhance the determination of the number of clusters, *k*, in the three-way k-means algorithm. The genetic algorithm was employed to learn the optimal k-value, leveraging its adaptive search and global optimization properties to identify a more stable set of initial clustering centers for k-means. This approach mitigated the issue of a low algorithm efficiency due to improper initial clustering center selection and enhanced the clustering results. Several key elements of this study’s methodology were as follows.

(1) Chromosome encoding: The encoding method for chromosomes played a pivotal role in determining the execution efficiency and search performance of genetic algorithms. In this study, an efficient binary encoding approach was employed to represent the *k*-value, utilizing an 8-bit binary number within the chromosome. The choice of encoding method significantly impacted the efficiency of algorithm execution and its overall search capabilities.
(2) Fitness function: Developed a fitness function to assess individual performance, directly influencing the algorithm’s convergence speed and its quest for the optimal solution. The fitness function employed in this context was based on the sum of squared distances, as presented in Formula (10).
(3) Selection operators: The selection operator, also referred to as the regeneration operator, serves the purpose of either directly passing on the optimized individuals to the next generation or creating new individuals through pairing and crossover before transmitting them to the subsequent generation. This study adhered to the “survival of the fittest” principle and employed the roulette wheel method to establish the probability of individual selection, which was directly contingent on their respective fitness values. In this method, the likelihood of an individual being chosen for offspring reproduction increased with a higher fitness value. The calculation for individual selection probability was as follows.
  (14) $$P\left(x_{i}\right)=\frac{f(x_{i})}{\sum_{j=1}^{n}f(x_{j})}$$
  Among them, $P(x_{i})$ represents the selection probability of the individual $x_{i}$, $f(x_{i})$ represents the fitness value of individual $x_{i}$, and *n* represents the number of individuals.
(4) Crossover operators: The crossover operator involves the random exchange of specific genes between two individuals within a population, determined by a designated crossover rate. This process aims to generate novel gene combinations with the expectation of combining favorable genes. The crossover operation was pivotal to the genetic algorithm, and the algorithm’s overall search capability heavily relied on it, ensuring global search effectiveness. In this study, a crossover operator was employed, and its crossover probability was automatically adjusted based on the fitness values of the chromosomes. When an individual’s fitness value fell below the average, a crossover operation was directly performed. Conversely, if an individual’s fitness value exceeded the average, a certain crossover probability was chosen. This approach not only preserved the traits of individuals with high fitness values, but also guaranteed that individuals with lower fitness values undergo crossover operations, further enhancing the algorithm’s global search capabilities. The calculation formula for an adaptive crossover probability was as follows.
  (15) $$P_{c}=\left\{\begin{array}{cc} \frac{f_{max}-f}{f_{max}-f_{avg}} & f\geq f_{avg}\\ 1 & f<f_{avg} \end{array}\right..$$
  Among them, $P_{c}$ represents the adaptive crossover probability, $f_{max}$ represents the maximum fitness value, $f_{avg}$ represents the average fitness value, and *f* represents the larger fitness value of the individual during the crossover operation.
(5) Mutation operators: The mutation operator serves the purpose of maintaining population diversity, preventing premature convergence, and introducing a localized random search capability. In this study, an adaptive probability-based mutation operator was implemented. When an individual’s fitness value exceeds the population average, a low mutation rate was applied to facilitate its transition to the next generation smoothly. Conversely, if an individual’s fitness value was below the average, a high-probability mutation operation was performed, increasing its chances of elimination. The calculation formula for adaptive mutation probability was as follows.
  (16) $$P_{m}=\left\{\begin{array}{cc} \frac{f_{max}-f}{f_{max}-f_{avg}} & f\geq f_{avg}\\ 1 & f<f_{avg} \end{array}\right..$$

Among them, $P_{m}$ represented the adaptive crossover probability, $f_{max}$ represented the maximum fitness value, $f_{avg}$ represented the average fitness value, and *f* represented the fitness value of the mutated individual.

For the implementation of the algorithms in this study, Python 3.8 was employed as the programming language of choice. The steps of the GA-based three-way k-means algorithm were shown in Algorithm 2.
**Algorithm 2** GA-based three-way k-means.**Input:** dataset *X* **Output:** three-way k-means result $C=\{\left(C_{o}\left(C_{1}\right),F_{r}\left(C_{1}\right)\right),...,\left(C_{o}\left(C_{k}\right),F_{r}\left(C_{k}\right)\right)\}$ 1:Divided the dataset *X* into kernel dataset K(X) and outer dataset R(X) by sample stability;2:Randomly generate an initial population of *k* individuals and compute the fitness values for each individual;3:After performing selection, crossover, and mutation operations, a new generation of the population was generated;4:Repeat steps 2 and 3 until the termination criteria were met, then conclude and output the optimal value of *k*;5:Based on the optimal value of *k*, use a genetic algorithm to perform the initial cluster center optimization for the kernel data set;6:Utilizing the optimal value of k and the optimal initial cluster centers, perform clustering on the kernel data set and update the cluster centers;7:Calculate the Euclidean distance between samples ∈K(X) and cluster centers;8:Assign the samples to the core region of the cluster that was closest to their respective cluster centers;9:Calculate the Euclidean distance between samples ∈R(X) and cluster centers;10:Assign the samples to the boundary region of the cluster that was closest to their respective cluster centers;11:Output the three-way k-means result.

### 2.7. Re-Clustering Strategy

The k-means model segmented samples by considering their Euclidean distances within the data set, designating the most suitable center point within each cluster as a potential monitoring location. Nevertheless, during the sensor placement process, practical factors must be taken into account, including considerations such as sunlight exposure sensors and the influence of fan-generated airflow, among other variables, apart from the temperature and humidity attributes. Consequently, the necessary number of monitoring points for each cluster might have differed, which underscores the rationale for incorporating re-clustering in this investigation.

In this study, a combination of the Three-Way Decision [28,29] and within-cluster similarity [30] was employed as an indicator to assess whether re-clustering was necessary. The formula for calculating the within-cluster similarity was as follows:

(17) $$IntraS\left(C_{m}\right)=\frac{2\times \sum_{i=1}^{|C_{m}-1|}\sum_{j>i}^{|C_{m}|}S_{A}\left(x_{mi},x_{mj}\right)}{|C_{m}\left|\times (\right|C_{m}\left|-1\right)}$$

where $C_{m}$ denoted the *m*-th class cluster in the set of class clusters, $x_{mi}$ denoted the *i*-th sample in the class cluster $C_{m}$, $x_{mj}$ denoted the *j*-th sample in the class cluster $C_{m}$, and $S_{A}\left(x_{mi},x_{mj}\right)$ denoted the similarity between the samples $x_{i}$ and $x_{j}$, which was calculated by the following formula:

(18) $$S_{A}\left(x_{mi},x_{mj}\right)=\frac{\sum_{i=1}^{\left|A\right|}I(x_{mil},x_{mjl})}{\left|A\right|}$$

where *A* denoted the set of attributes of the samples in the data set and $I(x_{mil},x_{mjl})$ denoted the similarity between the two samples, which was evaluated by comparing the individual attributes of the two samples and was calculated as follows:

(19) $$I\left(x_{mil},x_{mjl}\right)=\left\{\begin{array}{cc} i_{1} & x_{mil}=x_{mjl}\cap x_{i}\in C_{o}\left(C_{m}\right)\cap x_{j}\in C_{o}\left(C_{m}\right)\\ i_{2} & x_{mil}=x_{mjl}\cap x_{i}\in C_{o}\left(C_{m}\right)\cap x_{j}\in F_{r}\left(C_{m}\right)\\ i_{3} & x_{mil}=x_{mjl}\cap x_{i}\in F_{r}\left(C_{m}\right)\cap x_{j}\in F_{r}\left(C_{m}\right)\\ 0 & x_{mil}\neq x_{mjl} \end{array}\right..$$

(20) $$i_{1}+i_{2}+i_{3}=1$$

where $x_{mil}$ represented the *l*-th attribute of the *i*-th sample $x_{i}$ in cluster $C_{m}$, and $x_{mjl}$ represented the *l*-th attribute of the *j*-th sample $x_{j}$ in cluster $C_{m}$. When the values of $x_{mil}$ and $x_{mjl}$ were the same, and both sample $x_{i}$ and $x_{j}$ belong to the core domain $C_{o}\left(C_{m}\right)$ of cluster $C_{m}$, a score of $i_{1}$ was assigned. When the values of $x_{mil}$ and $x_{mjl}$ were the same, and sample $x_{i}$ belonged to the core domain $C_{o}\left(C_{m}\right)$ of cluster $C_{m}$, while sample $x_{j}$ belonged to the boundary domain $F_{r}\left(C_{m}\right)$ of the same cluster, a score of $i_{2}$ was assigned. When the values of $x_{mil}$ and $x_{mjl}$ were the same, and both sample $x_{i}$ and $x_{j}$ belonged to the boundary domain $F_{r}\left(C_{m}\right)$ of the cluster, a score of $i_{3}$ was assigned. If the values of $x_{mil}$ and $x_{mjl}$ were not the same, a score of 0 was assigned.

A higher within-cluster similarity implied a stronger relationship among samples within the cluster, resulting in improved clustering outcomes. Consequently, clusters with lower within-cluster similarity were identified as candidates for re-clustering. The selection of clusters requiring re-clustering involved calculating the average within-cluster similarity across all clusters and comparing it to the within-cluster similarity of each cluster. Clusters with a ratio exceeding 1 were designated for re-clustering. The final number of clusters post-reclustering was determined as the smallest integer greater than or equal to the ratio of the average within-cluster similarity to the within-cluster similarity of each cluster.

After completing the re-clustering procedure, the results from both clustering iterations were combined to derive the ultimate clustering result. In this final result, two sensors were picked from each cluster, including the cluster center, based on their proximity to this center. These chosen sensors constituted the pool of candidate sensors. Following this, one sensor was selected from each cluster, resulting in several sensor combinations.

### 2.8. Joint Entropy-Based Method (JEBM)

**Figure 4:**
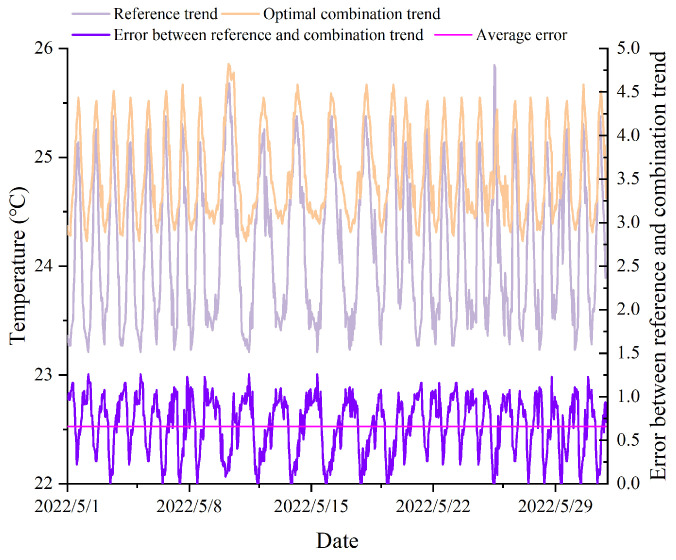
Example of reference trend and combination trend comparison (air temperature).

**Figure 5:**
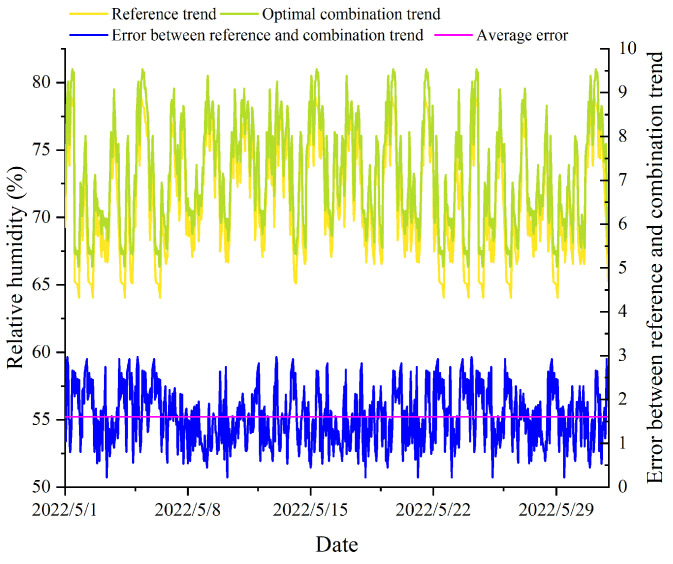
Example of reference trend and combination trend comparison (relative humidity).

Arnesano et al. [31] assumed that the average values obtained from all sensors placed inside a building could serve as a reference value representing the entire indoor environment of the building. In the case of sensor combinations derived through re-clustering, we calculated the average temperature and humidity values and compared them with the overall averages recorded within the pig house. This comparative analysis aided in the identification of sensor positions that could effectively monitor the overall environmental conditions of the pig house. Figure 4 and Figure 5 depicted a comparison between sensor combination measurements and the comprehensive environmental measurements gathered from within the entire pig house. The sensor combination comprised five sensors (L-8, L-9, L-18, L-22, L-23) strategically positioned at the central location inside the pig house. The “Reference trend” denoted the average values derived from all sensor monitoring data within the pig house. To assess the performance, we computed the error between the “Reference trend” and the “Combination trend” using the average values for each sensor combination. From the figures, it was evident that there existed a disparity between the selected sensor combination without a layout optimization and the overall pig house environment. The integration of the JEBM aimed to identify crucial sensor positions within the pig house, minimizing data detection inaccuracies.

To determine the optimal placement and quantity of sensors that effectively represented the overall environment within the pig house, a thorough assessment was conducted employing the joint entropy and clustering effectiveness indices. Information theory-based joint entropy was employed in this research to validate this hypothesis. Joint entropy, as defined by Shannon [32], quantifies the collective information content produced by multiple variables, and its formula was presented below.

(21) H(X,Y)=H(X)+h(Y/X)

(22) $$H\left(X\right)=\sum_{r=1}^{n}-P_{r}\left(E\right)\log_{2}P_{r}\left(E\right)$$

(23) $$H\left(Y/X\right)=-\sum_{x=1,j=1}^{m}P\left(x,y\right)\log\frac{P(x,y)}{P(x)}$$

(24) $$\sum_{i=1}^{m}T(X_{i})=H\left(X_{k}\right)+...+H\left(X_{l}\right)+\sum_{i=1}^{m-l}\sum_{j=k}^{l}H(X_{i},X_{j})$$

where H(X,Y) was the joint entropy of the temperature and relative humidity data measured by sensors, H(X) was the information entropy of the temperature data measured by sensors, H(Y/X) was the conditional entropy of the temperature and relative humidity data measured by sensors, $P_{r}\left(E\right)$ was the probability mass function for a variable, *r* was the range of the data (1 to n), $\sum_{i=1}^{m}T(X_{i})$ was the total entropy for the combinations of sensors, *m* was the total number of sensors in the pig house, *l* was the number of selected sensors, $H\left(X_{k}\right)+...+H\left(X_{l}\right)$ was the sum of the entropy values at the selected sensors, and $\sum_{i=1}^{m-l}\sum_{j=k}^{l}H(X_{i},X_{j})$ was the amount of information delivered from the unselected sensors to the selected sensors.

To assess the sensors in the core and boundary domains obtained through the three-way K-means clustering more effectively, this study integrated the Three-Way Decision approach with a clustering validity index [32]. As a result, we proposed the Three-Way Clustering Validity Index (TWCVI), with the formula provided as follows.

(25) TWCVI(C)=IntraS(C)−InterS(C)

where IntraS(C) denoted the average intra-class similarity of each class cluster; the larger the value meant the higher the similarity of the samples within the class cluster and the better the clustering effect; the calculation formula was as follows.

(26) $$IntraS\left(C\right)=\frac{\sum_{m=1}^{k}IntraS(C_{m})}{k}$$

where $IntraS(C_{m})$ denoted the intra-class similarity of cluster $C_{m}$; the calculation formula was shown in Formula (17).

InterS(C) represented the average inter-class similarity of each class cluster; the smaller the value indicates the lower the similarity of the samples between the class clusters and the better the clustering effect; it was calculated as follows.

(27) $$InterS\left(C\right)=\frac{2\times \sum_{m=1}^{k-1}\sum_{n<m}^{k}InterS(C_{m},C_{n})}{k\times (k-1)}$$

where *k* denotes the number of clusters, $InterS(C_{m},C_{n})$ denotes the inter-class similarity of class clusters and was calculated as follows.

(28) $$InterS\left(C_{m},C_{n}\right)=\frac{\sum_{i=1}^{|C_{m}|}\sum_{j=1}^{|C_{n}|}S_{A}\left(C_{mi},C_{nj}\right)}{|C_{m}\left|\times \right|C_{n}|}$$

where $C_{m}$ and $C_{n}$ denoted the *m*-th and *n*-th class clusters in the set of class clusters, respectively, $C_{mi}$ denoted the *i*-th sample in class cluster $C_{m}$, $C_{nj}$ denoted the *j*-th sample in class cluster $C_{n}$, and $S_{A}\left(C_{mi},C_{nj}\right)$ was the similarity between the two samples computed by Formula (18).

A higher value of TWCVI indicated that within the clustering results, individual clusters exhibit high within-cluster similarity, while simultaneously demonstrating low between-cluster similarity. This signifies better clustering performance.

From the information point of view, the optimal sensor placement should satisfy the basic objectives of maximizing the information content of all measurement points and maximizing the three-way clustering validity index.

### 2.9. Statistical Validation Metrics

The criteria for optimal sensor placement entailed the strategic selection of sensor monitoring positions that effectively represented the comprehensive internal environment of the pig house. This method leveraged the three-way k-means model and re-clustering to identify all potential sensor combinations. Subsequently, the joint entropy-based method (JEBM) was employed to assess these candidate sensor combinations, ultimately pinpointing the combination that best approximates the overall pig house environment. To validate the chosen optimal sensor placement, Root-Mean-Square Error (RMSE) and Mean Absolute Percentage Error (MAPE) values were used as evaluation metrics. Furthermore, a comparative analysis was conducted between the optimal sensor placement proposed in this paper and the solution selected by the reference model, offering empirical support for the efficacy of the model introduced in this study.

RMSE was a measure to evaluate the difference between the combination trend and reference trend, expressed in °C, and the formula was as follows.

(29) $$RMSE=\sqrt{\frac{\sum_{i=1}^{n}{\left(c_{i}-r_{i}\right)}^{2}}{n}}$$

MAPE was a percentage of the error and could be used to evaluate the accuracy relative to the reference value under each set of combinations, expressed in percentage (%), and the formula was as follows.

(30) $$MAPE=\frac{100\%}{n}\sum_{i=1}^{n}|\frac{c_{i}-r_{i}}{r_{i}}|$$

where *n* was the total number of data, $c_{i}$ was the value of the combination trend at a specific time, and $r_{i}$ was the value of the reference trend at a specific time.

### 2.10. Fitting Sensor Data from Optimal Sensor Placement Using Fuzzy Fusion

After optimizing the sensor layout, key sensors will be strategically positioned within the pigsty to effectively represent the overall environment. The collected data will serve as input for the control system. In many conventional designs, the data from the optimal sensor layout were typically averaged. Multiple data points in each dimension were combined into a single data point, which was then fed into the control system to regulate the pigsty’s internal environment. This approach was characterized by its simplicity and efficiency. However, it came with significant drawbacks, mainly the potential for errors due to variations in data recorded at different locations within the pigsty. For example, there could be notable temperature differences between data collected at ventilation vents and data collected at central points within the pigsty. Simple averaging at this stage could lead to substantial errors, compromising the control system’s accuracy.

To address this issue, we employed fuzzy logic to merge the data obtained from sensors in the optimal layout. This minimized errors, ensuring the provision of effective monitoring data for the control system and, consequently, the maintenance of a suitable environment within the pigsty.

Initially, our data set comprised temperature and humidity readings from various sensors strategically placed in the optimal sensor configuration. These sensors were situated at distinct locations, capturing environmental conditions from diverse points within the experimental setting. Nevertheless, these data points could be influenced by sensor inaccuracies, measurement noise, and potential disparities. Consequently, before amalgamation, we subjected the data to preprocessing, which encompassed correction, filtering, and handling of outliers to ensure the quality and consistency of the data.

Furthermore, we established relevant fuzzy sets based on the physiological characteristics of pigs. Considering the experimental context, which involved the fattening of pigs, the optimal temperature range for their growth fell within 17–23 °C. Therefore, we defined temperature categories as follows: low temperature (below 17.5 °C), moderate temperature (17.5–22.5 °C), and high temperature (above 22.5 °C). Similarly, with regard to humidity, the ideal range for fattening pigs in the experimental pens was 65–70%. As a result, we established humidity categories as dry (below 65.5%), suitable (65.5–69.5%), and humid (above 69.5%) [33].

Subsequently, after the formulation of these fuzzy sets, we employed Gaussian functions as membership functions to transform the temperature and humidity data into their respective fuzzy categories via the membership function. The Gaussian function has the property of smoothing and could effectively represent the degree of affiliation of the fuzzy set, which was reasonable for the fuzzification of temperature and humidity data. The selection of the appropriate fuzzy set corresponding to the detected temperature and humidity data was determined using voting theory. Voting theory can consider uncertainties and interrelationships between multiple fuzzy sets, which helps to improve the accuracy and robustness of fuzzy sets. This selected fuzzy set was then input into the control system, governing the regulation of the pigsty’s internal environment.

## 3. Results

### 3.1. Analysis of Data Inside the Experimental Pig House

**Figure 6:**
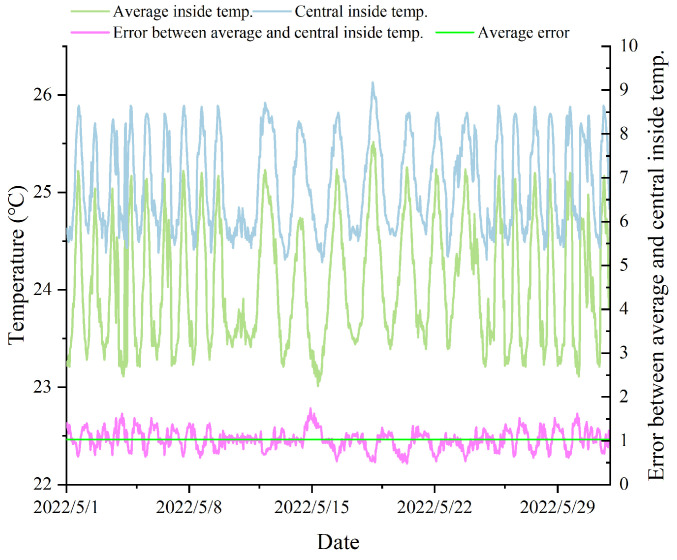
Comparison of average air temperature measured by all sensors inside the pig house and air temperature of centrally located sensor).

**Figure 7:**
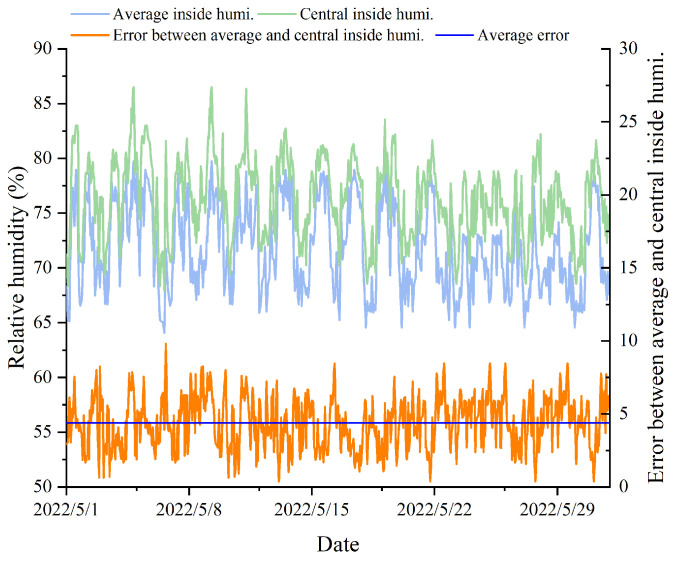
Comparison of average relative humidity measured by all sensors inside the pig house and relative humidity of centrally located sensor.

Figure 6 displays a comparison between the average temperature within the pig house during a specific period of the experiment and the measurements at the central location (1–31 May 2022). It can be concluded that the temperature at the central location within the pig house was higher than the average temperature representing the overall environment. Over a week of measurement data comparison, the central temperature was 1.74 °C higher at the maximum, 0.48 °C higher at the minimum, and averages 1.08 °C higher than the average temperature. The reason for this phenomenon may be that the sensors surrounding the central area were closer to the windows, fans, and wet curtains, resulting in less airflow in the central area and an accumulation of heat. Figure 7 illustrates a comparison between the average relative humidity and the measurements at the central location within the pig house during the same period. It was evident that the relative humidity data measured by the sensors located at the central position were higher than the average and exhibit larger fluctuations. The reason for this could be the presence of feeding troughs and water troughs on both sides of the pig pens, which were closer to the central area, leading to a higher relative humidity in the central location.

**Figure 8:**
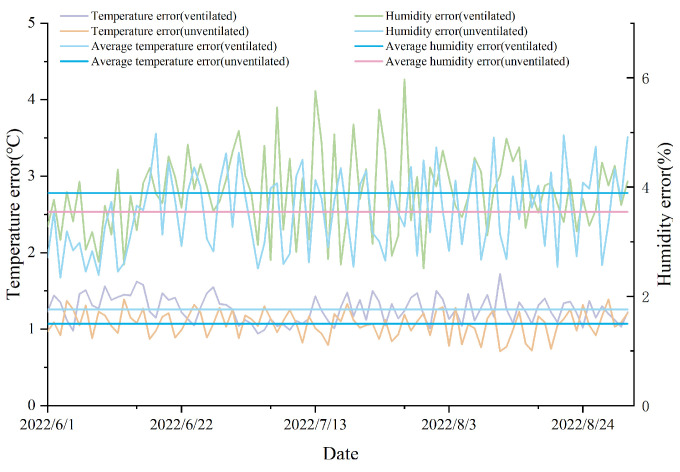
Time analysis of monitoring data fluctuations under different ventilation conditions.

During the experiment, the average indoor temperature in the pig house was recorded as 25.2 °C, with a maximum temperature of 30.4 °C and a minimum temperature of 20.7 °C. The highest temperature occurred around 14:10 on 7 July at location L-7. At this time, the indoor temperature was most affected by solar radiation, as L-7 was within the range of direct sunlight and farther away from the fans. Additionally, the lowest temperature was recorded by the sensor located at L-29 at around 22:35 on 29 September. The average humidity within the pig house during the experiment was 74.6%, with a maximum humidity of 88.5% and a minimum humidity of 63.7%. The highest humidity was observed around 07:05 on 21 September at location L-21, while the lowest humidity was recorded at around 15:50 on 9 July at location L-5. Throughout the entire experiment, variations in data were observed among sensors at different locations within the pig house due to varying levels of illumination and airflow. Among the temperature data collected by all the sensors, the maximum temperature difference was 4.6 °C, the minimum temperature difference was 1.1 °C, and the average temperature difference for each sensor was 2.8 °C. For humidity data, the maximum humidity difference was 9.8%, the minimum humidity difference was 7.4%, and the average humidity difference for each sensor was 7.9%. Furthermore, considering the presence of windows on the north and south sides, wet curtains on the east side, and fans on the west side of the pig house, sensors from L-1 to L-5 and L-11 to L-15 were significantly influenced by solar radiation and airflow during daytime temperature measurements. Sensors from L-21 to L-25 may have been more affected by the wet curtains, as well as feeding and water troughs within the pig pens when measuring humidity. We conducted a time analysis of the monitoring data fluctuations under different ventilation conditions, as illustrated in Figure 8.

Based on the above analysis, using data from sensors located in a single position or sensors significantly influenced by external factors to control the internal environment of the pig house may lead to improper control, resulting in suboptimal conditions for pig growth and imbalances in regional temperature and humidity. The effectiveness of sensor data was directly determined by the location of the sensors. When the data collected by sensors cannot represent the overall environment within the pig house, the maintenance of the rearing environment for the pigs becomes ineffective. Pigs were homeothermic animals and were highly sensitive to environmental temperature and humidity. To provide an appropriate growth environment for the pigs, it was necessary to select an optimal sensor placement that can represent the overall environment of the pig house. Furthermore, it was essential to recognize the substantial environmental variations that occur during different seasons, with particular emphasis on the pronounced differences between summer and winter. Consequently, this study centers its analysis on the data recorded during these two distinct seasons. It strategically devises dynamic sensor layouts within the pig pens tailored for both summer and winter conditions. The subsequent step involves merging these two optimized sensor configurations to derive a comprehensive and optimal sensor layout aptly suited for the entire duration of the experimental stage.

### 3.2. Evaluation of Optimal Sensor Placement

#### 3.2.1. Optimal Sensor Placement in Summer

Figure 8 displays the outcomes of assessing temperature and humidity sensor combinations in the experimental pig house during the summer months (June to August 2022) for varying quantities of sensors. The model presented in this study utilizes a PSO algorithm-based approach, incorporating re-clustering and JEBM, hereafter referred to as PSO_rj, while the control variables method serves as the basis for comparative evaluations. The notation PSO_r represents the model employing the three-way k-means algorithm based on the PSO algorithm with a re-clustering strategy, while PSO_j designates the model using the three-way k-means algorithm based on the PSO algorithm with JEBM. The notation Temp.J represents the joint entropy of the air temperature. The notation Temp.T represents the three-way clustering validity index of the air temperature. The notation Humi.J represents the joint entropy of relative humidity. The notation Humi.T represents the three-way clustering validity index of relative humidity. In the context of the three-way k-means validity index metric, both PSO_rj and PSO_r re-cluster the initial clustering results. Consequently, for the same number of clusters, the three-way k-means validity index metrics of these two models in the figure curves overlap.

**Figure 9:**
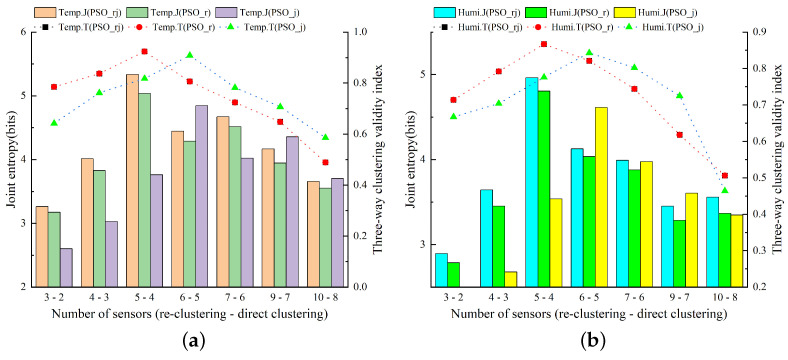
Evaluation of PSO-based three-way k-means model for optimal placement at different sensor numbers in summer (June to August). Temp.J—Joint entropy of air temperature; Temp.T—Three-way clustering validity index of air temperature; Humi.J—Joint entropy of relative humidity; Humi.T—Three-way clustering validity index of relative humidity: (**a**) Air temperature; (**b**) Relative humidity.

Figure 9a displays the results of assessing the optimal sensor combinations selected by the PSO-based three-way k-means model with air temperature data. The figure reveals that the number of clusters increases after re-clustering, in contrast to direct clustering without the re-clustering strategy. When evaluating the results based on the joint entropy, an assessment metric, the optimal performance was observed with five sensors. When analyzing the three-way clustering validity index metrics, both the PSO-based three-way k-means model with re-clustering and JEBM (PSO_rj) and the PSO-based three-way k-means model without re-clustering and with JEBM (PSO_j) both attain the maximum value of the evaluation index. When combining the two evaluation indexes, the PSO algorithm-based model proposed in this study consistently outperformed other sensor numbers, especially when the number of sensors was set to five. It achieved significant optimization compared to the comparison model. This suggests that with five sensors, the PSO algorithm-based model selects the sensor combination with the lowest error, making it a better representation of the overall air temperature environment inside the pigsty.

Figure 9b presents the evaluation results of optimal sensor combinations selected by the PSO-based three-way k-means model for humidity data. The figure illustrates the comparison of evaluation metrics for each model at varying numbers of sensors. It is evident from the figure that the sensor combination with five sensors provides the best representation of the relative humidity environment inside the pig house. Considering the results with air temperature data, the final number of sensors chosen for the PSO-based three-way k-means model in this experiment during summer was five.

Utilizing a single model for the sensor layout within the barn may have its limitations. To address this, we employed the GA-based three-way k-means model for sensor layout optimization. Figure 9 illustrates the outcomes of this approach in evaluating the combination of temperature and humidity sensors selected by the GA-based three-way k-means model in the experimental pig house during summer (June to August 2022). Consistent with the previously mentioned abbreviations and experimental framework, we designate GA_rj as the GA-based three-way k-means model developed in this study. GA_rj integrates the re-clustering strategy and JTBM (joint entropy-based method) after enhancing the three-way k-means algorithm using genetic algorithms. GA_r represents the model employing the GA-based three-way k-means algorithm with a re-clustering strategy. GA_j represents the model utilizing the GA-based three-way k-means algorithm with JEBM (joint entropy-based method). In terms of the three-way k-means validity index metric, the performance curves of the two models, GA_rj and GA_r, coincide.

**Figure 10:**
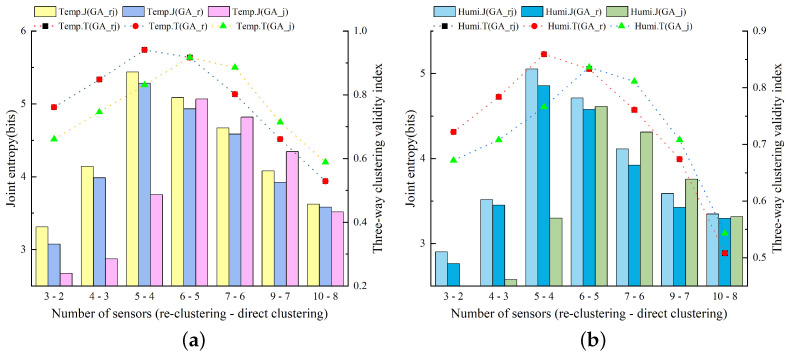
Evaluation of GA-based three-way k-means model for optimal placement at different sensor numbers in summer (June to August). Temp.J—Joint entropy of air temperature; Temp.T—Three-way clustering validity index of air temperature; Humi.J—Joint entropy of relative humidity; Humi.T—Three-way clustering validity index of relative humidity: (**a**) Air temperature; (**b**) Relative humidity.

Figure 10a depicts the results of evaluating the optimal sensor combinations selected by the GA-based three-way k-means model using air temperature data for different numbers of sensors. Regarding the evaluation using air temperature data, both the joint entropy and the three-way k-means validity index metrics reached their maximum value when the number of sensors was set to five. Furthermore, when experimentally compared with the comparison model, the GA-based three-way k-means model proposed in this study also demonstrated an optimal performance, affirming the effectiveness of the study’s design in enhancing the optimal sensor layout. Furthermore, when evaluating the metrics using relative humidity data, as shown in Figure 10b, the optimal result was also attained with a sensor count of five. This implies that the sensor combination selected with five sensors, capable of representing the overall temperature and humidity conditions inside the pig house, constitutes the optimal sensor layout within the current model.

Once the optimal sensor counts and positions were determined by the PSO-based three-way k-means model and the GA-based three-way k-means model, respectively, a comparative analysis was conducted to assess and contrast the resulting sensor arrangement schemes. Table 1 presents the sensor combinations selected for optimal sensor placement by both models, along with their respective RMSE and MAPE values. The optimal values were highlighted in bold. The table includes the mean values of temperature and humidity data recorded by these sensor combinations, with RMSE and MAPE serving as indicators of the average performance across the entire pigsty environment. By comparing Table 1 with Figure 2, it becomes evident that the sensor placements chosen by both models, exhibit similarities in terms of location, and both models, after improvement, have achieved a satisfactory performance. They were concentrated in the center of the experimental pig house and in the surrounding areas near the windows. This observation suggests that these positions were crucial within the experimental pig house, and situating sensors in these locations was an effective means of monitoring the overall environment inside the pig house and providing valuable data for subsequent control operations. The evaluation indexes, RMSE and MAPE, indicate that the errors associated with the sensor combinations chosen by both models were relatively small. This suggests that the data collected by these sensor combinations can effectively represent the comprehensive temperature and humidity environment within the pig house.

**Figure 11:**
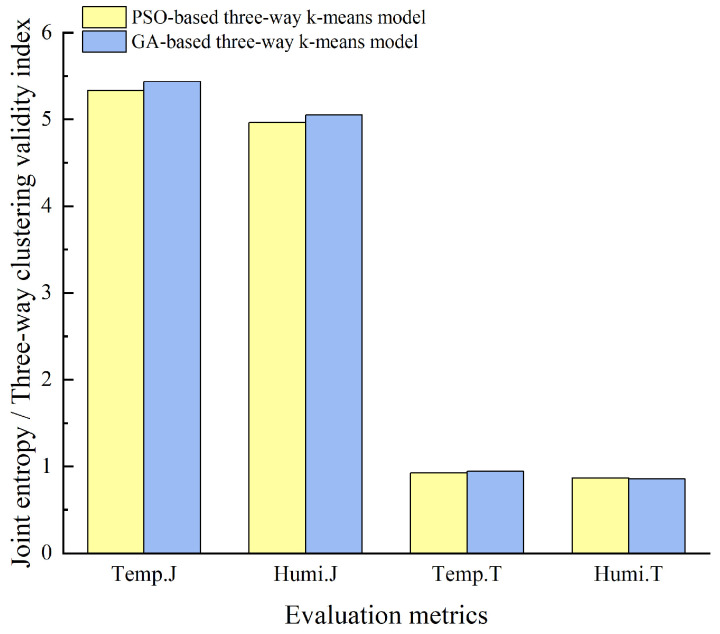
Evaluation of optimal placement for different models in summer (June to August). Temp.J: Joint entropy of air temperature; Humi.J: Joint entropy of relative humidity; Temp.T: Three-way clustering validity index of air temperature; Humi.T: Three-way clustering validity index of relative humidity).

Figure 11 provides a comparison of the optimal sensor placements chosen by the two models, evaluated using joint entropy and three-way clustering validity index. The figure illustrates that, overall, the GA-based three-way k-means model’s selected sensor placement performs slightly better. However, it was important to note that in the case of the three-way clustering validity index for relative humidity, the GA-based three-way k-means model performs marginally worse than the PSO-based three-way k-means model. Taking these findings into account, the sensor combination derived from the GA-based three-way k-means model was selected as the optimal sensor placement for the experimental pig house during the summer season.

#### 3.2.2. Optimal Sensor Placement in Winter

The most significant environmental differences within the year occur between summer and winter. During the summer, pig houses often require frequent window openings for ventilation, making sensor placement near these windows crucial. Conversely, in the winter, the environment tends to be more enclosed, and heating equipment may be in use, potentially shifting the key sensor locations to the center of the pig house and near the heating equipment, rather than close to the windows. As a result, the optimal sensor placement selected for summer data may not be suitable for the winter barn. Therefore, this study performed a separate optimization for the winter barn’s optimal sensor placement.

Figure 11 displays the outcomes of evaluating temperature and humidity sensor combinations selected by the PSO-based three-way k-means model for various numbers of sensors in the winter season (December 2021 to February 2022). The experimental setup paralleled the procedures performed during the summer months, employing the controlled variable method in both comparative experiments.

**Figure 12:**
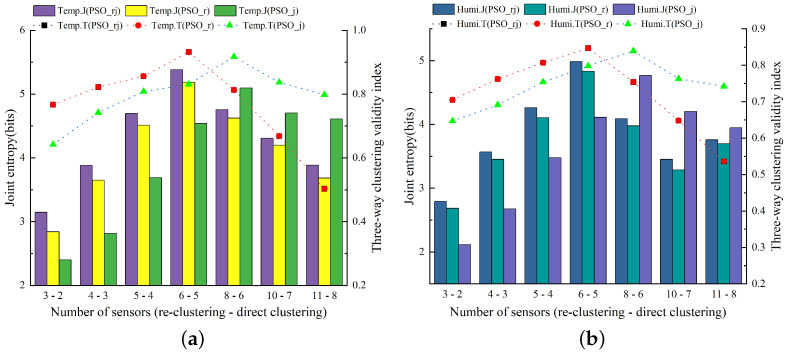
Evaluation of PSO-based three-way k-means model for optimal placement at different sensor numbers in winter (December to February). Temp.J—Joint entropy of air temperature; Temp.T—Three-way clustering validity index of air temperature; Humi.J—Joint entropy of relative humidity; Humi.T—Three-way clustering validity index of relative humidity: (**a**) Air temperature; (**b**) Relative humidity.

Figure 12a,b depict the assessment results for the optimal sensor combinations chosen by the PSO-based three-way k-means model for varying numbers of sensors in terms of the air temperature and relative humidity data, respectively. It was evident from the figures that the evaluation metrics for the sensor combinations selected by the model proposed in this study achieve optimal values when the number of sensors was set at six, outperforming the comparison models substantially. Consequently, the optimal number of sensors selected by the PSO-based three-way k-means model during the winter season was six, representing an increase in one sensor position in comparison to the layout used during the summer season.

**Figure 13:**
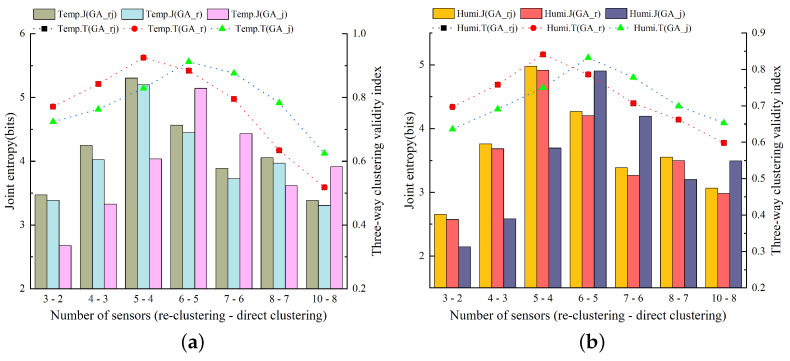
Evaluation of GA-based three-way k-means model for optimal placement at different sensor numbers in winter (December to February). Temp.J—Joint entropy of air temperature; Temp.T—Three-way clustering validity index of air temperature; Humi.J—Joint entropy of relative humidity; Humi.T—Three-way clustering validity index of relative humidity: (**a**) Air temperature; (**b**) Relative humidity.

Figure 13 presents the evaluation results of the optimal sensor combinations selected by the GA-based three-way k-means model with varying numbers of sensors in the experimental barn during the winter months (December 2021 to February 2022). Both Figure 13a,b illustrate the results of assessing the optimal sensor combinations for air temperature and relative humidity data, respectively.

Remarkably, at a sensor number of five, all the sensor combinations chosen by the model achieved optimal values under different sensor numbers. The GA-based three-way k-means model proposed in this study outperformed the two comparison models employing the control variable method. Consequently, the optimal number of sensors selected for sensor placement using the GA-based three-way k-means model during the winter season was five.

**Figure 14:**
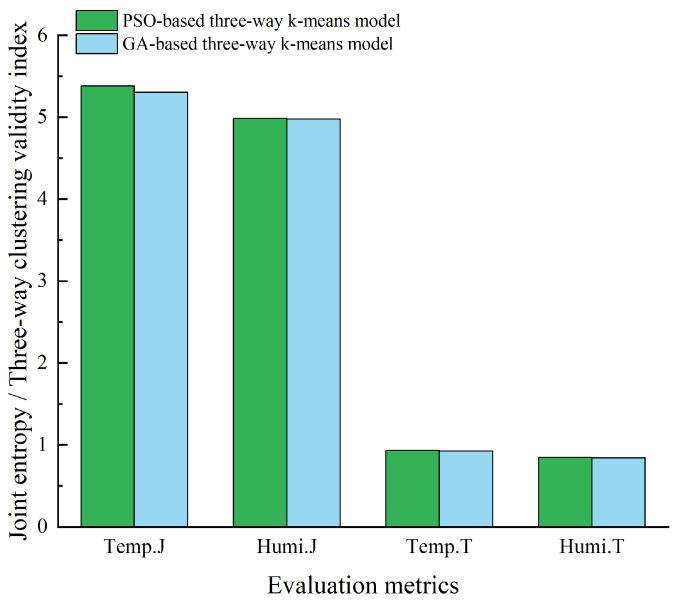
Evaluation of optimal placement for different models in winter (December to February). Temp.J: Joint entropy of air temperature; Humi.J: Joint entropy of relative humidity; Temp.T: Three-way clustering validity index of air temperature; Humi.T: Three-way clustering validity index of relative humidity.

Table 2 provides a comparative analysis of the sensor layout schemes selected by the PSO-based three-way k-means model and the GA-based three-way k-means model. The evaluation metrics of RMSE and MAPE were included, with the optimal values highlighted in bold. The table includes RMSE and MAPE values for the average temperature and humidity data measured by the sensor combinations; the smaller RMSE and MAPE values indicate a smaller error between the monitored data from the chosen sensor combinations and the actual environmental data inside the pigsty. This implies that our model performs better as the errors were minimized. Figure 14 illustrates a comparison of the optimal sensor placements chosen for each of the two models based on the evaluation metrics of joint entropy and three-way clustering validity index for reference and analysis. Considering the data in Table 2 and the results depicted in Figure 14, it was evident that the sensor combination chosen by the PSO-based three-way k-means model outperforms that selected by the GA-based three-way k-means model during winter. This could be attributed to the winter data being more suitable for the particle swarm optimization algorithm. The data closely aligns with the overall environment within the pig house. As a result, the sensor combination derived from the PSO-based three-way k-means model was deemed the optimal sensor placement for the winter season within the experimental pig house.

In summary, the dynamic optimal sensor placement model introduced in this study effectively estimates the overall environmental conditions within the experimental pig house during both summer and winter seasons, meeting the research objectives. A comparative analysis of the results from this model and the comparative models demonstrates that the optimal sensor placement from this study more accurately represents the overall barn environment, yielding smaller errors in the temperature and relative humidity that closely align with actual conditions. Thus, the approach outlined in this study proves to be effective and practical for monitoring the internal environment of the experimental pig house.

### 3.3. Evaluation of Dynamic Optimal Sensor Placement Fusion

In this study, a dynamic sensor layout was devised for the experimental barn to accommodate both summer and winter conditions. The experiments in this section aimed to assess environmental monitoring within the experimental barn using the sensor layout scheme generated by combining the optimal sensor layouts for both the summer and winter.

Table 3 presents the optimal sensor placements for the experimental pig house in summer, winter, and after the fusion. Combining the sensor layout schemes for summer and winter results in a total of seven sensors, which will be evaluated in the following sections.

Table 4 shows the experimental evaluation when the experimental pig barn was monitored using the fused sensor layout. Recorded in the table were the RMSE and MAPE of the average of the temperature and humidity data measured by the sensor combination versus the average representing the overall environment of the pig house. The table reveals that the fused sensor layout exhibits relatively minor errors in the temperature and humidity data gathered across all four seasons, satisfying the design specifications and effectively representing the overall temperature and humidity environment inside the experimental pig house. While the performance in summer and winter may not be as exceptional as the sensor layout scheme before fusion, the reduction in accuracy remains within acceptable limits. Hence, experimental verification has demonstrated the feasibility of the sensor layout scheme achieved by dynamically selecting optimal sensor placements for both summer and winter seasons. This layout effectively fulfills the requirements of accurately characterizing the overall temperature and humidity environment within the experimental pig house. The ultimate decision to dynamically adjust the number and location of sensors in the summer and winter or adopt the fused sensor layout scheme should be made according to the specific circumstances of the experimental pig house.

### 3.4. Evaluation of Fuzzy Fusion

In this study, we assessed the performance of the optimal sensor layout during summer, the optimal sensor layout during winter, and the sensor layout fused from both summer and winter using Fuzzy Fusion throughout the entire experimental period, which includes spring, summer, fall and winter. The assessment involved a comparison of the temperature and humidity data monitored by the sensors in each layout, followed by processing with Fuzzy Fusion. These processed results were then compared with the overall internal environment in the pig house to validate the efficacy of Fuzzy Fusion.

Table 5 presents the assessment results of the air temperature data monitored by each sensor layout after applying Fuzzy Fusion. The optimal sensor layout for summer was determined using the GA-based three-way k-means model, resulting in the selection of a total of five sensors (L-2, L-8, L-10, L-19, L-28). The optimal sensor layout for winter was derived using the PSO-based three-way k-means model, with a total of six sensors selected (L-2, L-8, L-10, L-22, L-24, and L-28). The optimal sensor layouts for summer and winter were integrated to create a sensor layout suitable for the entire experimental phase, involving a total of seven sensors (L-2, L-8, L-10, L-19, L-22, L-24, and L-28). The term “overall temperature” signifies the temperature representing the comprehensive environment within the barn, and it was determined by averaging the measurements obtained from all the sensors within the barn. This average was computed using data from all the sensors in the barn.

For each season during the experimental phase, one day was chosen for a comparative analysis. In the legend, the symbol “-” signifies that the sensor layout was not tested during that particular time period. The term “Low” corresponds to Fuzzy Fusion output indicating a low temperature (below 17.5 °C), “Moderate” represents Fuzzy Fusion output for a moderate temperature (17.5–22.5 °C), and “High” indicates Fuzzy Fusion output for a high temperature (above 22.5 °C).

The accuracy of the Fuzzy Fusion results, evaluated over the entire experimental phase (September 2021–September 2022), reached 98.2%. However, it was worth noting that most misclassifications occurred at critical temperature thresholds. For instance, on 13 October 2021, at 18:00, the Fusion optimal sensor placement indicated a low temperature, whereas the average temperature inside the barn was 17.6 °C, which was in close proximity to the critical value. This proximity to critical thresholds can lead to misclassifications. In summary, Fuzzy Fusion effectively fulfills the initial design requirements for sensor data processing, providing dependable data inputs to the control system. This ensures that the control system receives valuable information and can regulate the internal environment of the pig house effectively.

Table 6 presents the evaluation results of the relative humidity data monitored by individual sensor layouts after Fuzzy Fusion. “Overall humidity” represents the relative humidity characterized the overall barn environment and was determined by averaging measurements from all the sensors within the pig house.

For each season of the experimental phase, one day was selected for a comparative display. In the table, “-” indicates that the sensor layout was not tested during that period, “Dry” signifies that Fuzzy Fusion output indicated low humidity (below 65.5%), “Suitable” denotes that Fuzzy Fusion output indicated moderate humidity (65.5–69.5%), and “Humid” represents Fuzzy Fusion output indicating high humidity (above 69.5%).

The results of Fuzzy Fusion were validated using the optimal sensor placement fused throughout the experimental phase (September 2021 to September 2022), achieving an accuracy rate of 96.9%. This level of accuracy aligns with the design requirements and underscores the effectiveness of Fuzzy Fusion.

## 4. Discussion

This study proposes a dynamic sensor layout scheme based on the varying environmental requirements within a pigsty across different seasons, aiming to achieve a rational and effective monitoring system for pigsties. The principal contributions of this endeavor were delineated as follows:

(1) We optimize the dynamic sensor layout within the pigsty, implementing distinct sensor placement strategies for different seasons (summer and winter). This approach ensures a rational and effective monitoring of the pigsty’s internal environment, thus providing valuable monitoring data for optimal control within the pig house.
(2) We employed both the three-way k-means model based on the particle swarm optimization algorithm and the three-way k-means model based on the genetic algorithm to develop the optimal sensor layout scheme. After conducting comprehensive experimental comparisons and analyses, we determined the final optimal sensor placement within the experimental pig house.
(3) We apply the three-way k-means algorithm to cluster sensors. After clustering, we select sensors that were located near the cluster center within each cluster to form candidate sensor combinations. We then choose the best solution from these candidate sensor combinations. This approach resolves the issues related to high computational costs and inefficiency when iterating through all possible sensor combinations.
(4) We employ a fuzzy fusion method to handle the data acquired by the chosen optimal sensor layout scheme. The temperature and humidity data collected by the sensors undergo fuzzification using fuzzy sets and membership functions, and the results were determined through a voting theory. This approach prevents significant errors that might affect the control system, as can be the case with simple averaging.
(5) We integrated the optimal sensor placements from both summer and winter and confirmed that the fused sensor layout accurately represents the comprehensive temperature and humidity conditions within the experimental pigsty.

In summary, the exploration of three-way clustering algorithms for sensor placement remains relatively unexplored in the existing literature. This study introduces a novel approach by employing a three-way k-means model and a re-clustering strategy for sensor clustering, followed by the utilization of a joint entropy-based method to assess and determine the optimal sensor placement scheme for deployment in the experimental pigsty. This study differs from similar research in that it employs two commonly used algorithms in the sensor optimization layout to establish models, conducting a comparative analysis to choose the optimal one. Additionally, the study dynamically adjusts the layout with seasonal variations considered in the sensor optimization layout. Furthermore, a fuzzy fusion strategy was applied to the monitoring data instead of simple averaging, aiming to reduce errors introduced by other factors such as light and airflow. Nevertheless, this study has certain limitations. Firstly, in monitoring internal temperature and humidity within pigsties, the impact of wind speed was not incorporated, leading to a neglect of its influence on heat stress in pigs. Secondly, due to variations in the architectural structures of livestock sheds, the applicability of our model is somewhat limited. Therefore, our future research will focus on integrating Computational Fluid Dynamics (CFD) simulations to better account for airflow effects and enhance the model’s applicability.

## 5. Conclusions

Ensuring the effective monitoring of the overall internal environment of a pig house and providing an optimal growth environment for the pigs hinges on the careful selection of sensor quantity and placement. Presently, in existing pigsties, sensors were commonly installed based on the designer’s experience, which can lead to reduced monitoring efficiency and accuracy. This study has introduced a dynamic sensor layout optimization method that allows for the efficient and cost-effective monitoring of the pig house’s overall environment, providing essential data for the microclimate control system.

In this study, we enhanced the k-means model using PSO and GA algorithms, conducting an experimental sensor optimization layout for a pigsty. The improved model yielded optimal sensor configurations for different seasons, providing a rational and effective monitoring solution while reducing computational costs and enhancing operational efficiency. The experimental results substantiate the feasibility of the seasonal dynamic sensor layout concept. The optimized sensor layout achieved RMSE and MAPE values for temperature and humidity data below 0.28, meeting the predefined experimental objectives. This ensures the rational effectiveness of the monitored data, offering comprehensive data support for the operation of the control system. Despite achieving favorable experimental results in the pigsty, this study currently has certain limitations and shortcomings: (1) In optimizing the sensor layout within the experimental pigsty, the impact of indoor air flow on the monitoring effectiveness was not fully considered. This oversight may result in local effects on sensors due to vortices or eddies present within the pigsty. Although we employed fuzzy fusion in the study to mitigate the impact of similar anomalous situations, further investigations were warranted. (2) The study only monitored temperature and humidity data, without exploring the concentration monitoring of harmful gases such as carbon dioxide and ammonia. However, when creating an optimal living environment for pigs to enhance their welfare, monitoring the concentration of harmful gases is crucial. This opens up new avenues for our future research. (3) The current model is specifically designed for pigsties and has not demonstrated broad applicability. Addressing this limitation will be a key focus of our future research efforts.

## Figures and Tables

**Table 1:** Different sensor placements obtained from the two models in summer.

Models	Sensor Placements	RMSE (Temp.)	RMSE (Humi.)	MAPE (Temp.)	MAPE (Humi.)
PSO-based three-way k-means model	L-4, L-7, L-14, L-17, L-28	0.269	**0.275**	0.192	0.207
GA-based three-way k-means model	L-2, L-8, L-10, L-19, L-28	**0.237**	0.294	**0.172**	**0.178**

**Table 2:** Different sensor placements obtained from the two models in winter.

Models	Sensor Placements	RMSE (Temp.)	RMSE (Humi.)	MAPE (Temp.)	MAPE (Humi.)
PSO-based three-way k-means model	L-2, L-8, L-10, L-22, L-24, L-28	0.248	**0.266**	**0.184**	**0.211**
GA-based three-way k-means model	L-2, L-8, L-10, L-22, L-24	**0.227**	0.285	0.207	0.228

**Table 3:** Comparison of different sensor arrangement schemes.

Schemes	Sensor Placements	Sensor Numbers
Summer	L-2, L-8, L-10, L-19, L-28	5
Winter	L-2, L-8, L-10, L-22, L-24, L-28	6
Fusion	L-2, L-8, L-10, L-19, L-22, L-24, L-28	7

**Table 4:** Evaluation of fused sensor placement.

Seasons	RMSE (Temp.)	RMSE (Humi.)	MAPE (Temp.)	MAPE (Humi.)
Autumn (September 2021–November 2021 )	0.256	0.283	0.188	0.208
Winter (December 2021–February 2022)	0.238	0.263	0.194	0.206
Spring (March 2022–May 2022)	0.251	0.285	0.198	0.211
Summer (June 2022–August 2022)	0.246	0.289	0.172	0.188

**Table 5:** Air Temperature evaluation results of Fuzzy Fusion.

Date	Optimal Sensor Placement in Summer	Optimal Sensor Placement in Winter	Fusion Optimal Sensor Placement	Overall Temperature
Autumn (23 October 2021 11:00)	-	-	Moderate	21.3 °C
Winter (23 January 2022 11:00)	-	High	High	22.7 °C
Spring (23 April 2022 11:00)	-	-	Low	17.2 °C
Summer (23 July 2022 11:00)	Moderate	-	Moderate	22.1 °C

**Table 6:** Relative humidity evaluation results of Fuzzy Fusion.

Date	Optimal Sensor Placement in Summer	Optimal Sensor Placement in Winter	Fusion Optimal Sensor Placement	Overall Temperature
Autumn (23 October 2021 11:00)	-	-	Suitable	67.3%
Winter (23 January 2022 11:00)	-	Dry	Dry	65.1%
Spring (23 April 2022 11:00)	-	-	Suitable	68.6%
Summer (23 July 2022 11:00)	Humid	-	Humid	69.8%

## Data Availability

The data that support the findings of this study are available on request from the corresponding authors.
